# Patient Safety Subcultures among Nursing Home Staff in Italy: A Cross-Sectional Study

**DOI:** 10.3390/healthcare11131962

**Published:** 2023-07-07

**Authors:** Ilaria Tocco Tussardi, Lucia Cazzoletti, Maria Elisabetta Zanolin, Annarita Comini, Donatella Visentin, Emanuele Torri, Stefano Tardivo, Francesca Moretti

**Affiliations:** 1 Department of Diagnostics and Public Health, Section of Hygiene, University of Verona, 37134 Verona, Italy; 2 Department of Diagnostics and Public Health, Section of Epidemiology and Medical Statistics, University of Verona, 37134 Verona, Italy; 3 Department of Prevention, Healthcare Trust of the Autonomous Province of Trento, 38123 Trento, Italy; 4 Clinical Governance Service, Healthcare Trust of the Autonomous Province of Trento, 38123 Trento, Italy; 5 Department of Neurosciences, Biomedicine and Movement, University of Verona, 37134 Verona, Italy

**Keywords:** healthcare provider, nursing home, Nursing Home Survey on Patient Safety Culture, long-term care, patient safety, professional subcultures

## Abstract

Nursing home (NH) residents are vulnerable subjects and highly susceptible to adverse events. Knowledge of patient safety culture (PSC) is essential for an organization to ensure patient safety. However, research on PSC in NHs, and its variability among staff, is still scarce. This study aimed to explore whether and how PSC differed among NH staff (Managers, Nurses, Direct Care Staff, Support Staff, Administrative Staff and Other Providers) in the Autonomous Province of Trento, Italy. This study employed a cross-sectional design and collected data from 1145 NH providers using the Nursing Home Survey on Patient Safety Culture (NHSPSC). Data were analyzed using linear mixed models, with each of the 12 NHSPSC domains as a response variable. The majority of the respondents (61.6%) were Direct Care Staff members. ‘Feedback and Communication about Incidents’ and ‘Overall Perceptions of Resident Safety’ were the domains with the highest proportions of positive answers (PPAs). For most staff categories, ‘Staffing’ was the domain with the lowest PPA. Support Staff showed significantly lower scores in the majority of domains (8/12). Shorter job tenure, fewer weekly working hours, working mostly during the day and working in highly specialized areas were associated with higher scores in several domains. Interventions to improve PSC must consider the differences between professional groups. Further research is needed to explore the relationship between job-related features and perceptions of patient safety among NH workers.

## 1. Introduction

In healthcare settings, ensuring safety is a cornerstone for providing adequate care. As the attention paid to patient safety increases [1], studies indicate that a considerable number of adverse events in healthcare are preventable [2,3]. There is growing evidence to support that prevention strategies are effective at improving safety levels [4].

In this respect, safety culture is an essential concept for healthcare organizations to strengthen patient safety and quality of care. Patient safety culture (PSC) is a subset of organizational culture and influences patient safety [5]. It is essential to understand these underlying cultural factors within an organization, as the culture of safety impacts residents’ care experiences, as well as healthcare workers’ well-being.

Several studies have investigated PSC at the organizational and individual levels in different healthcare settings. Among these, nursing homes (NHs) are still regarded as a critical context in terms of the provision of quality and patient safety [6,7,8]. The risk of errors in the delivery of care is particularly high, and this is primarily (even if not entirely) related to the characteristics of residents. Multi-morbidity, multi-therapy, the need for high integration between different specialities, functional dependency and cognitive impairment put this population at high risk of adverse events [9,10,11]. Several studies have highlighted the importance of promoting PSC among NH workers, and evidence links better safety culture to improved quality-of-care measures (e.g., fewer falls and pressure ulcers, and less restraint use) [3,12,13,14].

Knowledge of what type of safety culture exists within an organization is a prerequisite to guiding change. A thorough assessment using validated tools allows for the identification of strengths and weaknesses, raises awareness of safety issues among staff and constitutes the basis of developing tailored interventions for improvement and to follow up change [15]. Moreover, assessing PSC can highlight a factor that might hinder improvement and is peculiar to multi-professional organizations: the existence of safety subcultures. It is acknowledged that the culture of safety naturally differs between departments, specialties and professional groups [16,17,18]. However, if the subcultures are not aligned with organization-wide safety goals, then patient safety might be at risk. Despite the relevance of this issue, there is still a limited number of studies addressing how PSC differs between professions. Moreover, only a few studies have focused on PSC among NH staff. A scoping review by Gartshore et al. retrieved a relatively low number of texts exploring safety culture in care homes for older people (n = 25), and there was a lack of studies from European countries (2 out of 25, specifically from the Netherlands and Switzerland) [1].

Knowledge of PSC and its determinants among NH staff is fundamental to achieve safe patient care in such a delicate setting, and the role played by these professionals will grow increasingly in the context of an aging population. In 2021, 23.4% of the total population in Italy was 65 years and older, with 1830 NH residents aged ≥ 65 years for 100,000 inhabitants [19]. The aim of this study was to explore whether and how PSC differed among NH staff in a northern Italian region. To the best of our knowledge, this is the first study to investigate professional PSC in the Italian NH setting.

## 2. Materials and Methods

### 2.1. Study Design

This cross-sectional study was conducted on a convenience sample of 25 NHs in the Autonomous Province of Trento, Italy (45.4% of all NHs of the province, n = 55). To assess the PSC among NH staff, the Italian version of the Nursing Home Survey on Patient Safety Culture (NHSPSC) validated by Moretti et al. (under submission) was used. The survey was planned according to the Strengthening the Reporting of Observational Studies in Epidemiology (STROBE) guidelines and the User Guide for the NHSPSC of the Agency for Healthcare Research and Quality (AHRQ) [20]. Further details on the NHSPSC and data collection procedures are described below.

### 2.2. Setting

The Autonomous Province of Trento is an alpine territory in northeastern Italy, with an area of 6207.12 sq. km, a population of almost 550,000 inhabitants and a life expectancy at birth that was 81.6 years for males and 86.3 years for females in 2019 [21]. In 2020, the number of beds in NHs per 1000 inhabitants aged ≥ 65 years was more than two times greater than the national average (38.8 vs. 15.0, respectively) [21]. NH care is delivered through the public system. Residents are mostly elderly people. NHs provide their residents with 24 h assistance, including nursing and personal care, medical care and rehabilitation, and occupational and social activities. Some NHs have dementia care units or special care units that are designed, staffed and equipped to provide care for people suffering from cognitive impairment or other complex clinical conditions requiring enhanced care.

### 2.3. Survey Instrument

The NHSPSC is designed specifically for NH providers and asks for their opinions about the culture of patient safety and the healthcare quality in their NHs [20]. The survey consists of 42 items and 12 composite measures grouping two or more items that assess the same area of culture (i.e., domains). Additional measures include overall ratings and background questions. An agreement scale and frequency scale are used as part of the NHSPSC. The agreement scale uses the following: Strongly Agree, Agree, Neither, Disagree, and Strongly Disagree. The frequency scale uses the following: Always, Most of the Time, Sometimes, Rarely, and Never. The answer Not Applicable/Don’t Know is also included.

### 2.4. Data Sources and Study Participants

Data were collected between late 2018 and early 2019. A total of 2478 paper copy of the NHSPSC were distributed to the staff of 25 NHs (2368 beds in total). In accordance with the AHRQ User Guide [20], we established a system-level point of contact (D.V.), and each NH established a point of contact among staff members to support the survey administration, maintain open communication throughout the project and provide with assistance. Publicity material was created to announce and promote the survey in the NHs. The local points of contact were responsible for distributing the surveys to NH staff. Staff members were informed of the aim of the study, data confidentiality and the voluntary nature of the participation. No monetary incentives were offered to complete the survey. Surveys were returned to locked drop boxes placed throughout the NHs. The system-level point of contact was responsible for monitoring survey returns, in collaboration with local point of contacts, and for stimulating participation where needed.

Overall, 1224 workers completed the survey. The average response rate for the 25 NHs was 59% (ranging from 31 to 90%). Of the 1224 respondents, 1145 indicated their professions and were considered for the present study. Staff categories were defined in accordance with the original version of the NHSPSC as Administrators/Managers (which included administrators, medical directors and directors of nursing; physicians were also included in this staff category due to their low number); Administrative Support Staff (which included administrative assistants, admissions, billing, secretaries and human resources); Nurses; Direct Care Staff (which included nursing assistants/aides, healthcare technicians and physical therapists); Support Staff (which included personnel not working directly with residents (i.e., driving service, food service, dietary, housekeeping, laundry service and maintenance)); and Other Providers (which included staff having limited contact with residents (i.e., dietitians, nutritionists, occupational therapists, speech therapists, respiratory therapists, social workers and psychologists)). Table 1 shows the number of surveys returned and the composition of respondents, by staff category. The composition of the sample reflects the average composition of NH staff at the regional level [22].

### 2.5. Analyses

Descriptive statistics included the proportion of positive answers (PPA) for the individual categories of staff for each survey item and domain. The PPA was chosen as the outcome measure in accordance with the NHSPSC 2019 User Database Report [23]. ‘Agree/Strongly Agree’ or ‘Most of the Time/Always’ responses for positively worded items and ‘Disagree/Strongly Disagree’ or ‘Rarely/Never’ responses for negatively worded items were considered as positive responses. The domain score consisted of the average proportion of positive answers for all items included in the domain. Data were analyzed for the six different staff categories described above (Administrators/Managers, Administrative Support Staff, Nurses, Direct Care Staff, Support Staff and Other Providers).

Linear mixed models were used for the score analyses of the 12 NHSPSC domains, with each domain as a response variable, the staff type and other background characteristics as the effects of interest (length of time working in the NH; hours per week usually working in the NH; usual work shift; paid by a staffing agency when working for the NH; and usual area or unit of work) and a facility random effect to account for the clustering effect. For the analyses, we used 100-point-scale domain scores, which were calculated according to the following formula [24]:

Domain score = (average of items’ scores* − 1) × 25

Analyses were conducted using Stata software, version 16 (StataCorp. 2019. Stata Statistical Software: Release 16. College Station, TX: StataCorp LLC).

* On Likert scale.

## 3. Results

The majority of the respondents (61.6%) were Direct Care Staff members. The highest proportion of respondents (45.9%) had been working in their current NHs for 11 years or more and the majority (71.9%) worked in their NHs for 25–40 h per week.

The PPA by staff type for the items and domains are given in Table 2. For Administrators/Managers, the highest PPA were for the items ‘Staff are told right away when there is a change in a resident’s care plan’ and ‘Staff are given all the information they need to care for residents’. For Nurses, Direct Care Staff and Support Staff, the highest PPA was for the item ‘Staff tell someone if they see something that might harm a resident’. For Administrative Support Staff, the highest PPA was for the item ‘This nursing home is a safe place for residents’. For Other Providers, the highest PPA was for the item ‘Residents are well cared for in this nursing home’. For all the staff categories, the item with the lowest PPA was ‘Staff have to hurry because they have too much work to do’.

For Administrators/Managers, Nurses and Support Staff, the domain with the highest PPA was ‘Feedback and Communication about Incidents’, while for Direct Care Staff, Administrative Support Staff and Other Providers, it was ‘Overall Perceptions of Resident Safety’. The domain with the lowest PPA was ‘Staffing’ for the majority of categories (Administrators/Managers, Nurses, Administrative Support Staff and Other Providers), while for Direct Care and Support Staff, it was ‘Non-punitive Response to Mistakes’.

Table 3 reports the results of the application of linear mixed models to the domain scores for the staff category and variables of interest. The staff categories Administrators/Managers and Administrative Support Staff scored higher than the reference category (Nurses) for the domains ‘Staffing’ (*p* < 0.001 for Administrators/Managers), ‘Communication Openness’ (*p* = 0.005 for Administrators/Managers) and ‘Management Support for Resident Safety’ (*p* = 0.001 for Administrators/Managers and *p* = 0.026 for Administrative Support Staff). Direct Care Staff showed mixed results compared to the reference category, with significantly higher scores than the reference group for the domain ‘Staffing’ (*p* = 0.012), and significantly lower scores for the domains ‘Handoffs’ (*p* = 0.007), ‘Communication Openness’ (*p* = 0.022) and ‘Supervisor Expectations and Actions Promoting Resident Safety’ (*p* = 0.012). Support Staff showed significantly lower scores on average than the reference group in the majority of domains (8 out of 12).

Staff who had been working in their current NHs for at least 3 years scored significantly lower than the reference group (staff with less than 2 months of seniority) in 7 out of 12 domains. For some domains, differences from the reference category were already evident at 2–11 months of seniority (the domains ‘Teamwork’ and ‘Training and Skills’). From 1–2 and 3–5 years of seniority, differences also became significant for the domains ‘Staffing’, ‘Supervisor Expectations and Actions Promoting Resident Safety’, ‘Overall Perceptions of Resident Safety’, ‘Management Support for Resident Safety’ and ‘Organizational Learning’. For the domain ‘Management Support for Resident Safety’, the difference with the reference category increased progressively with seniority. Interestingly, for some domains, the peak difference to the reference was reached in the middle seniority bands (for ‘Staffing’, ‘Training and Skills’, ‘Supervisor Expectations and Actions Promoting Resident Safety’, ‘Overall Perceptions of Resident Safety’ and ‘Organizational Learning’).

Staff working for at least 16 h per week in their NHs showed a significantly lower score on average for the domain ‘Teamwork’ (*p* = 0.006, *p* = 0.008 and *p* = 0.015 for 16–24 h, 25–40 h and >40 h per week, respectively) compared to providers working 15 h or less.

Staff working mainly during the night shift scored significantly lower on average on ‘Teamwork’ (*p* < 0.001), ‘Non-punitive Response to Mistakes’ (*p* < 0.001), ‘Feedback and Communication about Incidents’ (*p* = 0.003) and ‘Communication Openness’ (*p* = 0.005) compared to staff working during the daytime (reference group).

Staff working mainly in areas or units dedicated to the care of residents affected by cognitive impairment (Alzheimer’s/dementia) showed significantly higher scores on average for ‘Teamwork’ (*p* = 0.001), ‘Staffing’ (*p* = 0.023) and ‘Management Support for Resident Safety’ (*p* = 0.043) compared to providers not working in a specialized area/unit (reference group).

Finally, being paid by a staffing agency did not entail significant differences in any domain.

## 4. Discussion

This cross-sectional study on over 1000 NH workers is the first to address PSC in long-term care settings in Italy and to focus on differences among professional groups.

From the items that recorded the highest PPA among the group Administrators/Managers (‘Staff are told right away when there is a change in a resident’s care plan’ and ‘Staff are given all the information they need to care for residents’), we could outline that the management had a very positive perception of the quality of communication among frontline workers, especially with regard to the planning of care. This was in line with the perception of safety and high standards of care expressed by Administrative Support Staff and providers having limited contact with residents (the items ‘This nursing home is a safe place for residents’ and ‘Residents are well cared for in this NH’). The groups Administrators/Managers and Administrative Support Staff had the highest proportions of positive responses in most domains, and this finding was in line with the results of several previous studies [8,15,23,25,26].

With regard to the differences between administration and bedside staff, an interesting finding was the high rating given by Administrators/Managers and Administrative Support Staff to ‘Management Support of Resident Safety’, which was not confirmed by the other professional categories. This was in line with the significantly higher scores expressed by Administrators/Managers and Administrative Support Staff compared to the reference category in the multivariate analysis. This misalignment could indicate that managers were perceived of as detached from frontline staff’s safety. It can be assumed that management is oriented towards ensuring safety through the standardization of practices and the development of procedures, while frontline workers, who face safety issues on a daily basis, feel the need to implement different resources (e.g., non-technical skills). As mentioned above, the alignment of leadership with frontline staff is crucial for effectively promoting safety [16,17,18], as well as for accountability and successful clinical governance in nursing homes following the COVID-19 pandemic [27].

With regard to the domains related to communication, good proportions of positive responses were observed with regard to communication about adverse events (‘Feedback and Communication about Incidents’) by various staff categories. These results were in line with those of the study by Desmedt et al. [26] and the 2019 AHRQ surveys on PSC conducted on 10,499 NH providers [23]. However, the multivariate analysis showed a more negative perception by all categories of frontline workers than that expressed by Nurses, with a significant difference for Support Staff.

Another area of criticality that emerges from the results of the frontline workers concerns the ‘Non-punitive response to errors’, where Support Staff scored significantly lower than Nurses, who scored lower than Administrators/Managers (yet not significantly). These data may denote the perception of hierarchies among professional categories. The significantly lower scores on ‘Communication Openness’ by Direct Care Staff compared to Nurses, who scored significantly lower than Administrators/Managers, could confirm this hypothesis. Such settings may find it difficult to implement safety projects, whereas a proper PSC treats errors and problems as opportunities for improvement. This attitude at the practice level should be supported by NH management [28,29].

In terms of the proportion of positive responses, the staffing of NHs was rated below the percentage threshold of sufficiency by all categories (<50%), but the Support Staff group, in particular, showed a significantly lower score than the reference group. These data are in line with the results of some previous studies in the long-term care setting [30,31] and are a relevant finding for patient safety. Indeed, the perception of inadequate staffing for care demands is related to the perception of poor quality of care [32,33,34,35] and may lead to feelings of frustration among personnel, with an increased likelihood of errors and severe consequences for both residents/patients [33,35,36,37] and staff [38,39]. This situation can be exacerbated when the workload significantly exceeds the available capacity, as happened during the COVID-19 pandemic [40,41].

Another relevant aspect concerns the results of ‘Handoffs’. In particular, from the multivariate analysis, a significantly lower perception emerged from Direct Care Staff compared to Nurses. This is an important aspect because these are the two categories with the most direct contact with residents, and a perception of a lack of involvement in care seems to emerge. This may have potentially negative consequences for the effectiveness of multidisciplinary teams.

The focus on the involvement of the whole team also emerged from the results of the multivariate analysis for ‘Supervisor Expectations and Actions Promoting Resident Safety’. Again, Direct Care Staff, as well as Support Staff, scored significantly lower than Nurses, highlighting the perception that limited attention is being paid to the frontline staff and an area for improvement.

With regard to the results from the multivariate analyses showing significantly lower scores for Support Staff for most domains, we could hypothesize that staff who may not hold solid safety culture tools (e.g., non-healthcare backgrounds) are inclined to judge situations more severely, as also suggested by the results from Titlestad et al. [42]. This leads to a reflection on the appropriateness of extending projects to improve PSC among staff of all categories.

The analysis also showed that employees with at least three years of seniority scored significantly lower than those with a minimum tenure (less than two months) in the majority of domains. This finding is in line with that of the 2019 AHRQ Report, in which respondents who had worked in NHs for less than one year had the highest average percentages of positive responses in the NHSPSC domains, whereas respondents who had worked in NHs from three to five years had the lowest [23]. It can be assumed that staff who have been working longer are less motivated and might be more aware of the weaknesses of the organization. Because it is unknown why such factors affect staff perspectives on safety practices, more qualitative and in-depth research is required to further explore the reasons behind individual differences in safety culture scores related to work tenure. Specific events or interactions may cause those with longer tenures to rate the safety culture less highly.

Regarding differences in the safety culture ratings among workers working different shifts, our results are in line with those of the 2019 AHRQ Report, where day-shift staff had the highest scores when averaging all domains, and night-shift staff had the lowest [23]. It can be hypothesized that providers who work at night perceive a less safe situation for residents, perhaps because of the reduction in staff during the night, or because some issues that are kept under control during the day are exacerbated during the night, when management options are limited, and staff are sometimes less experienced.

Finally, the analyses showed better patient safety ratings among workers in specialist areas. This could be explained by the better organization and staffing of these units due to the higher criticality of the patients, resulting in better work efficiency and the perception of safety.

### Limitations and Strengths

The study was conducted on a limited number of NHs, and the results may not be representative of the entire long-term care setting in Italy. The 25 NHs constituted a convenience sample, and this could be a source of research bias. The response rate, although globally adequate, was not satisfactory for all the participating NHs. The NHs were part of the same geographical, administrative and organizational environment, and we are aware that, even within the same country, there are significant regional variations in the characteristics of residential care. Finally, it should be noted that the survey captures the safety climate and thus reflects staff opinion, and therefore comparisons must consider subjectivity.

However, this study also presents some strengths. Although this was a convenience sample, the sample size allows for the reasonable generalization of the results and the extension of the actions for improvement to a broader context than the one investigated. In addition, the heterogeneity of the sample adds further value to the data collected. Another strength of the study is that we followed a strong and validated methodology.

## 5. Conclusions

Measuring the PSC in an organization is an effective method for identifying actions that focus on safety improvements. In this context, management plays a key role, as both a cultural reference and a process guidance.

This study shows that Italian NHs face challenges similar to those reported by international studies, but some seem to be more specific to this context. Attention to staffing, improvement in the relationship with operators involved in adverse events and the better engagement of frontline operators have emerged as fundamental actions to improve the well-being of workers and safety for users.

## Figures and Tables

**Table 1:** Number of surveys returned and composition of respondents, by staff category (n = 1145).

Staff Category	Number of Surveys Returned	Percentage of Respondents
Direct Care Staff	705	61.6%
Nurses	167	14.6%
Other Providers	80	7%
Administrators/Managers ^1^	74	6.5%
Support Staff	67	5.8%
Administrative Support Staff	52	4.5%
Total	1145	

^1^ Physicians were included in Administrators/Managers category due to their low number (n = 2).

**Table 2:** NHSPSC (Nursing Home Survey on Patient Safety Culture) proportions of positive answers (%) by staff category.

Domain and Item *	Admin/Managers	Licensed Nurses	Direct Care Staff	Admin Support Staff	Support Staff	Other Providers
1. Teamwork	61.2	53.6	49.2	69.3	43.2	50.6
A1. Staff in this nursing home treat each other with respect.	63.9	53.7	46.3	72.5	39.7	46.2
A2. Staff support one another in this nursing home.	64	61.2	52.9	84	43.8	53.8
A5. Staff feel like they are part of a team.	49	40.7	41.8	54	34.4	41
A9. When someone gets really busy in this nursing home, other staff help out.	68	58.9	56	66.7	54.8	61.3
2. Staffing	47.2	36.5	38.7	46.4	36.6	40.1
A3. We have enough staff to handle the workload.	37	20	20.1	47.9	23.3	27.8
A8. Staff have to hurry because they have too much work to do. (N)	29	12.9	15.4	31.8	12.7	26.3
A16. Residents’ needs are met during shift changes.	72.7	68.4	76.1	60	79.5	55.7
A17. It is hard to keep residents safe here because so many staff quit their jobs. (N)	50	44.7	43.3	45.9	30.8	50.7
3. Compliance with Procedures	57.4	59.2	62.9	55.7	52.4	66.5
A4. Staff follow standard procedures to care for residents.	88	80	81.5	82.2	72.5	85.5
A6. Staff use shortcuts to get their work done faster. (N)	38.6	42.2	48.3	37.5	37.3	56.5
A14. To make work easier, staff often ignore procedures. (N)	45.6	55.4	59	47.4	47.3	57.4
4. Training and Skills	60.6	59.2	59.7	66.6	58.7	63.5
A7. Staff get the training they need in this nursing home.	79	71.7	70.2	79.6	61.2	74.7
A11. Staff have enough training on how to handle difficult residents.	39.1	46	45.6	69.2	53.3	54.1
A13. Staff understand the training they get in this nursing home.	63.6	60	63.4	51.1	61.7	61.6
5. Non-punitive Response to Mistakes	51.3	44.1	38.5	54.4	29.3	46.3
A10. Staff are blamed when a resident is harmed. (N)	46.4	42.2	29.5	50	20.5	46
A12. Staff are afraid to report their mistakes. (N)	41.4	39.1	42.2	41	30	42.3
A15. Staff are treated fairly when they make mistakes.	65.2	50.3	38.5	72.7	34	56.7
A18. Staff feel safe reporting their mistakes.	52.2	44.6	43.8	53.8	32.7	40.3
6. Handoffs	85.5	76	67.9	78.7	54.9	75.2
B1. Staff are told what they need to know before taking care of a resident for the first time.	88	77.6	66.9	75.9	54.1	79.5
B2. Staff are told right away when there is a change in a resident’s care plan.	90	79	67.4	77.3	50	73
B3. We have all the information we need when residents are transferred from the hospital.	74	67.3	61.7	73.1	51.7	64
B10. Staff are given all the information they need to care for residents.	90	80.1	75.7	88.6	63.6	84.2
7. Feedback and Communication about Incidents	86.5	79.5	73.9	78.3	77.4	71.9
B4. When staff report something that could harm a resident, someone takes care of it.	88	85	69.8	83.9	82.5	67.6
B5. In this nursing home, we talk about ways to keep incidents from happening again.	85	70.8	69.5	74.3	69.8	73.3
B6. Staff tell someone if they see something that might harm a resident.	88	89.6	88	81.3	85.7	76.3
B8. In this nursing home, we discuss ways to keep residents safe from harm.	85	72.7	68.6	73.7	71.4	70.5
8. Communication Openness	69.8	52.7	44.8	54.9	45.3	53.9
B7. Staff ideas and suggestions are valued in this nursing home.	71	49.4	39.2	52.4	40.3	40.5
B9. Staff opinions are ignored in this nursing home. (N)	60.3	42.6	36.1	37.2	44.9	56.6
B11. It is easy for staff to speak up about problems in this nursing home.	78	66	59.2	75	50.8	64.5
9. Supervisor Expectations and Actions Promoting Resident Safety	79.7	74.8	68.7	84.5	64	81.8
C1. My supervisor listens to staff ideas and suggestions about resident safety.	85	78.3	66.7	73.8	64.4	82.4
C2. My supervisor says a good word to staff who follow the right procedures.	69	61.6	58.1	84.1	56.7	74.7
C3. My supervisor pays attention to safety problems in this nursing home.	86	84.7	81.3	95.7	70.9	88.3
10. Overall Perceptions of Resident Safety	84.7	76.9	75	92.8	76.4	84.6
D1. Residents are well cared for in this nursing home.	89	82.8	81	95.5	82.5	91.3
D6. This nursing home does a good job keeping residents safe.	76	73.8	70.8	85.4	74.2	77.5
D8. This nursing home is a safe place for residents.	89	74.2	73.1	97.6	72.6	85
11. Management Support for Resident Safety	65.3	40.3	41.1	66.3	46.9	58
D2. Management asks staff how the nursing home can improve resident safety.	66	42.9	44.5	60.5	52.2	60.5
D7. Management listens to staff ideas and suggestions to improve resident safety.	75	47.1	44.8	72.5	53.1	61.3
D9. Management often walks around the nursing home to check on resident care.	55	30.8	33.8	65.9	35.4	52.1
12. Organizational Learning	69.3	60	57	71.8	63.4	63.9
D3. This nursing home lets the same mistakes happen again and again. (N)	72.9	60.8	60.5	65.1	66.7	62.7
D4. It is easy to make changes to improve resident safety in this nursing home.	54	45.6	43.8	51.4	48.2	54.5
D5. This nursing home is always doing things to improve resident safety.	74	69.8	63.6	87.5	73.7	69.2
D10. When this nursing home makes changes to improve resident safety, it checks to see if the changes worked.	76.5	63.7	60	83.3	65.1	69.3

* Average proportions of positive answers are given (i.e., Agree/Strongly Agree or Most of the Time/Always for positively worded items, and Disagree/Strongly Disagree or Rarely/Never for negatively worded items). The domain score consisted of the average proportion of positive answers for all items included in the composite measure. The intra-questionnaire average non-response rate was 6.6%. N: negatively worded items.

**Table 3:** Linear mixed models for NHSPSC domain score analyses. Coefficients (with 95% CI, *p*-value) for the association between domain scores and variables of interest. For brevity, domains are indicated with the progressive numbers reported in Table 2.

Domain Reference Number												
	1	2	3	4	5	6	7	8	9	10	11	12
Staff category												
Nurses *												
Admin/ Managers	5.31 (−0.48;11.10) *p* = 0.072	10.76 (5.13;16.39) *p* < 0.000	−1.35 (−7.33;4.61) *p* = 0.656	0.75 (−4.97;6.48) *p* = 0.796	3.61 (−2.19;9.42) *p* = 0.222	4.87 (−0.98;10.73) *p* = 0.103	0.71 (−5.11;6.53) *p* = 0.810	8.97 (2.75;15.18) *p* = 0.005	4.03 (−1.56;9.62) *p* = 0.158	4.20 (−0.74;9.14) *p* = 0.096	11.65 (4.67;18.62) *p* = 0.001	4.74 (−0.37;9.87) *p* = 0.069
Direct Care Staff	−3.16 (−6.81;0.47) *p* = 0.089	4.54 (1.00;8.08) *p* = 0.012	0.43 (−3.31;4.19) *p* = 0.819	−0.61 (−4.17;2.95) *p* = 0.736	−3.17 (−6.83;0.47) *p* = 0.088	−5.00 (−8.64;−1.35) *p* = 0.007	−3.06 (−6.68;0.55) *p* = 0.097	−4.52 (−8.39;−0.64) *p* = 0.022	−4.46 (−7.96;−0.96) *p* = 0.012	0.73 (−2.36;3.84) *p* = 0.640	0.76 (−3.60;5.12) *p* = 0.732	−1.42 (−4.64;1.79) *p* = 0.386
Admin Support Staff	4.58 (−2.12;11.28) *p* = 0.180	3.33 (−3.18;9.84) *p* = 0.316	2.07 (−4.91;9.07) *p* = 0.560	2.73 (−3.82;9.29) *p* = 0.414	6.06 (−0.75;12.89) *p* = 0.082	4.26 (−3.66;12.20) *p* = 0.292	−4.63 (−12.12;2.86) *p* = 0.226	0.21 (−7.01;7.44) *p* = 0.954	4.83 (−1.67;11.34) *p* = 0.146	4.83 (−1.32;10.99) *p* = 0.124	9.78 (1.15;18.40) *p* = 0.026	3.39 (−2.82;9.61) *p* = 0.285
Support Staff	−13.12 (−19.96;−6.28) *p* < 0.000	−6.73 (−13.36;−0.10) *p* = 0.047	−7.81 (−14.94;−0.68) *p* = 0.032	−7.00 (−13.69;−0.31) *p* = 0.040	−11.06 (−17.93;−4.20) *p* = 0.002	−7.40 (−15.07;0.25) *p* = 0.058	−11.31 (−18.41;−4.22) *p* = 0.002	−5.99 (−13.17;1.19) *p* = 0.102	−13.13 (−19.87;−6.38) *p* < 0.000	−7.06 (−13.02;−1.11) *p* = 0.020	−3.83 (−12.24;4.58) *p* = 0.372	−6.03 (−12.21;0.13) *p* = 0.055
Other Providers	−5.68 (−11.65;0.28) *p* = 0.062	3.78 (−2.00;9.57) *p* = 0.200	4.40 (−1.88;10.69) *p* = 0.170	−2.08 (−7.92;3.75) *p* = 0.484	−2.56 (−8.72;3.60) *p* = 0.415	−2.29 (−15.07;0.) *p* = 0.454	−5.64 (−11.61;0.32) *p* = 0.064	−4.13 (−10.49;2.21) *p* = 0.202	−3.94 (−9.74;1.86) *p* = 0.183	0.79 (−4.29;5.88) *p* = 0.759	8.19 (1.00;15.38) *p* = 0.026	−2.90 (−8.18;2.36) *p* = 0.280
Job tenure												
<2 m *												
2–11 m	−8.83 (−17.26;−0.40) *p* = 0.040	−1.92 (−9.96;6.12) *p* = 0.639	−2.00 (−10.58;6.57) *p* = 0.647	−8.52 (−16.77;−0.27) *p* = 0.043	−2.94 (−11.24;5.35) *p* = 0.486	0.42 (−8.02;8.87) *p* = 0.922	−0.19 (−8.55;8.16) *p* = 0.964	−2.75 (−11.82;6.31) *p* = 0.551	−2.63 (−10.59;5.31) *p* = 0.516	−2.05 (−9.12;5.02) *p* = 0.569	−5.75 (−15.68;4.17) *p* = 0.256	−4.15 (−11.50;3.20) *p* = 0.269
1–2 y	−12.74 (−21.10;−4.39) *p* = 0.003	−6.48 (−14.45;1.48) *p* = 0.111	−5.59 (−14.06;2.87) *p* = 0.195	−14.35 (−22.53;−6.17) *p* = 0.001	−6.19 (−14.35;1.96) *p* = 0.137	−4.76 (−13.10;3.56) *p* = 0.262	−3.08 (−11.35;5.18) *p* = 0.465	−7.52 (−16.51;1.45) *p* = 0.100	−9.25 (−17.15;−1.35) *p* = 0.022	−7.74 (−14.74;−0.74) *p* = 0.030	−12.72 (−22.52;−2.91) *p* = 0.011	−9.41 (−16.67;−2.15) *p* = 0.011
3–5 y	−13.84 (−22.09;−5.58) *p* = 0.001	−9.29 (−17.16;−1.41) *p* = 0.021	−6.30 (−14.69;2.08) *p* = 0.141	−9.44 (−17.52;−1.36) *p* = 0.022	−3.42 (−11.48;4.63) *p* = 0.405	−6.55 (−14.79;1.68) *p* = 0.119	−4.30 (−12.48;3.88) *p* = 0.303	−7.08 (−15.99;1.82) *p* = 0.119	−8.76 (−16.57;−0.95) *p* = 0.028	−9.94 (−16.87;−3.01) *p* = 0.005	−12.66 (−22.37;−2.95) *p* = 0.011	−10.72 (−17.91;−3.52) *p* = 0.003
6–10 y	−15.23 (−23.36;−7.11) *p* < 0.000	−8.08 (−15.82;−0.34) *p* = 0.041	−9.06 (−17.28;−0.83) *p* = 0.031	−14.60 (−22.55;−6.65) *p* < 0.000	−6.23 (−14.15;1.69) *p* = 0.123	−5.68 (−13.80;2.42) *p* = 0.170	−3.35 (−11.41;4.70) *p* = 0.414	−10.34 (−19.10;−1.57) *p* = 0.021	−13.85 (−21.51;−6.20) *p* < 0.000	−9.62 (−16.42;−2.82) *p* = 0.006	−14.91 (−24.43;−5.38) *p* = 0.002	−11.32 (−18.38;−4.27) *p* = 0.002
≥11 y	−12.90 (−20.77;−5.04) *p* = 0.001	−9.05 (−16.54;−1.56) *p* = 0.018	−9.16 (−17.12;−1.20) *p* = 0.024	−11.57 (−19.28;−3.85) *p* = 0.003	−8.40 (−16.06;−0.74) *p* = 0.032	−3.57 (−11.42;4.28) *p* = 0.373	0.96 (−8.77;6.84) *p* = 0.809	−9.82 (−18.30;−1.33) *p* = 0.023	−8.07 (−15.48;−0.67) *p* = 0.032	7.75 (14.34;1.16) *p* = 0.021	−15.98 (−25.21;−6.75) *p* = 0.001	−9.49 (−16.32;−2.66) *p* = 0.006
Work hours per week												
≤15 *												
16–24	−13.46 (−23.15;−3.77) *p* = 0.006	−5.16 (−14.58;4.25) *p* = 0.282	−1.09 (−11.11;8.91) *p* = 0.830	−2.24 (−11.73;7.24) *p* = 0.642	−4.05 (−14.36;6.26) *p* = 0.441	−1.19 (−10.90;8.50) *p* = 0.809	4.91 (−4.71;14.54) *p* = 0.317	−3.19 (−13.42;7.03) *p* = 0.541	−1.92 (−11.23;7.38) *p* = 0.685	2.52 (−5.75;10.81) *p* = 0.550	−3.73 (−15.32;7.86) *p* = 0.528	−0.33 (−8.93;8.25) *p* = 0.938
25–40	−12.90 (−22.37;−3.43) *p* = 0.008	−5.50 (−14.71;3.69) *p* = 0.241	−0.88 (−10.67;8.89) *p* = 0.859	−3.07 (−12.34;6.19) *p* = 0.516	−4.64 (−14.72;5.43) *p* = 0.367	−4.54 (−14.02;4.93) *p* = 0.347	1.96 (−7.45;11.37) *p* = 0.683	−5.58 (−15.57;4.41) *p* = 0.274	−4.18 (−13.28;4.91) *p* = 0.368	1.03 (−7.06;9.13) *p* = 0.802	−7.44 (−18.79;3.89) *p* = 0.198	−2.85 (−11.25;5.54) *p* = 0.505
>40	−13.82 (−24.98;−2.66) *p* = 0.015	−8.62 (−19.46;2.22) *p* = 0.119	3.22 (−8.30;14.75) *p* = 0.583	−2.53 (−13.45;8.38) *p* = 0.649	−5.78 (−17.41;5.85) *p* = 0.330	−10.79 (−22.05;0.46) *p* = 0.060	0.78 (−10.33;11.90) *p* = 0.889	−7.30 (−19.14;4.53) *p* = 0.226	−6.00 (−16.77;4.76) *p* = 0.274	−1.72 (−11.26;7.80) *p* = 0.722	−6.83 (−20.18;6.51) *p* = 0.315	−5.11 (−15.00;4.76) *p* = 0.310
Most frequent shift												
Day *												
Afternoon/Evening	−0.31 (−3.72;3.08) *p* = 0.856	−3.05 (−6.36;0.25) *p* = 0.070	1.56 (−1.94;5.08) *p* = 0.382	2.95 (−0.38;6.29) *p* = 0.082	−2.24 (−5.65;1.16) *p* = 0.196	−2.72 (−6.13;0.68) *p* = 0.117	−1.74 (−5.13;1.63) *p* = 0.311	−5.05 (−8.66;−1.43) *p* = 0.006	−4.22 (−7.50;−0.95) *p* = 0.011	−1.54 (−4.45;1.36) *p* = 0.297	−3.41 (−7.48;0.64) *p* = 0.100	−1.22 (−4.24;1.78) *p* = 0.425
Night	−33.55 (−49.06;−18.03) *p* < 0.000	−9.27 (−24.33;5.79) *p* = 0.228	2.77 (−13.22;18.76) *p* = 0.734	−9.88 (−25.07;5.29) *p* = 0.202	−27.77 (−43.19;−12.35) *p* < 0.000	−5.51 (−20.95;9.93) *p* = 0.484	−22.97 (−38.32;−7.61) *p* = 0.003	−23.54 (−39.88;−7.20) *p* = 0.005	−11.57 (−26.45;3.31) *p* = 0.128	−9.51 (−22.75;3.72) *p* = 0.159	−16.90 (−35.45;1.65) *p* = 0.074	−9.37 (−23.10;4.35) *p* = 0.181
Paid by a staffing agency												
Yes *												
No	0.14 (−3.74;4.04) *p* = 0.942	0.90 (−2.84;4.65) *p* = 0.636	0.69 (−3.28;4.67) *p* = 0.731	0.33 (−3.52;4.19) *p* = 0.864	0.33 (−3.57;4.24) *p* = 0.867	−1.19 (−5.10;2.72) *p* = 0.550	2.84 (−1.06;6.75) *p* = 0.154	−3.21 (−7.17;0.75) *p* = 0.113	0.26 (−3.40;3.93) *p* = 0.887	−1.34 (−4.71;2.01) *p* = 0.433	−3.23 (−7.95;1.47) *p* = 0.178	−0.67 (−4.12;2.78) *p* = 0.703
Area/unit of work ^1^												
Not specified *												
Alzheimer’s/Dementia	8.76 (3.65;13.87) *p* = 0.001	5.77 (0.81;10.73) *p* = 0.023	3.47 (−1.79;8.74) *p* = 0.196	4.76 (−0.29;9.83) *p* = 0.065	4.65 (−0.42;9.73) *p* = 0.073	2.98 (−2.10;8.08) *p* = 0.251	0.43 (−4.63;5.50) *p* = 0.865	3.98 (−1.42;9.38) *p* = 0.149	2.52 (−2.37;7.42) *p* = 0.313	3.24 (−1.11;7.60) *p* = 0.145	6.30 (0.18;12.42) *p* = 0.043	3.29 (−1.22;7.82) *p* = 0.153
Skilled nursing	−3.84 (−8.74;1.05) *p* = 0.124	−6.77 (−11.52;−2.01) *p* = 0.005	−2.62 (−7.67;2.42) *p* = 0.308	−8.80 (−13.63;−3.97) *p* < 0.000	−1.53 (−6.39;3.33) *p* = 0.537	−4.48 (−9.36;0.39) *p* = 0.072	−1.81 (−6.67;3.03) *p* = 0.463	−4.28 (−9.43;0.85) *p* = 0.102	−4.59 (−9.28;0.10) *p* = 0.055	−4.73 (−8.92;−0.55) *p* = 0.026	−4.80 (−10.69;1.09) *p* = 0.110	−3.79 (−8.13;0.53) *p* = 0.086
Other	−4.63 (−12.78;3.50) *p* = 0.264	−8.26 (−16.16;−0.35) *p* = 0.041	−0.98 (−9.37;7.41) *p* = 0.819	−10.68 (−18.83;−2.54) *p* = 0.010	−14.33 (−22.76;−5.90) *p* = 0.001	−7.53 (−15.84;0.77) *p* = 0.076	−7.89 (−16.15;0.35) *p* = 0.061	−6.45 (−15.18;2.28) *p* = 0.148	−5.31 (−13.12;2.50) *p* = 0.183	−8.01 (−15.10;−0.91) *p* = 0.027	−7.77 (−17.78;2.24) *p* = 0.128	−5.78 (−13.19;1.62) *p* = 0.126

* Reference category. ^1^ The NHSPSC answer ‘Rehab unit’ is not presented as it was not chosen by any respondent. Respondents answered the survey referring to their current NHs. NHSPSC background question n. 6, ‘In your job in this nursing home, do you work directly with residents most of the time?’, was not considered in the analysis. m = months; y = years.

## Data Availability

The data presented in this study are available upon reasonable request to the corresponding author.
